# Huge hypertrophic scar secondary to chronic ingrown toe nail mimicking tumor: A case report

**DOI:** 10.1097/MD.0000000000037005

**Published:** 2024-01-19

**Authors:** Baruenchan Ju, Young Sang Lee, Dong Hee Kim, Kwang-Bok Lee

**Affiliations:** a Department of Orthopaedic Surgery, Soo Hospital, Jeonju, Korea; b Department of Orthopaedic Surgery, Research Institute of Clinical Medicine of Jeonbuk National University – Biomedical Research Institute of Jeonbuk National University Hospital, Jeonbuk National University Medical School, Jeonju, Korea.

**Keywords:** hypertrophic scar, ingrowing nail, keloids

## Abstract

**Rationale:**

A huge hypertrophic scar formation secondary to chronic ingrown toe nail mimicking tumor is a rare disease. It is not only causing concerns cosmetically, but also hindering normal daily activities physically and socially. In this paper, we present an unusual case of bilateral ingrown nails with different phases. One resulted in a large hypertrophic scar caused by stimulation from secondary to chronic ingrown nail.

**Patient concerns and diagnosis:**

A 44-year-old man with a huge mass (7 × 4 × 8.5 cm) in his right great toe and inflamed ingrown nail in his left great toe visited the clinic. The mass in the right toe showed an irregular and bizarre shape with a stellate ulcer (2 × 2 cm) at the distal end. After removing an ingrown nail 3 years ago with minor repetitive trauma, self-managed wound has grown into a tumor-like mass, resulting in intolerable discomfort. In gross appearance, a stalk appeared to originate from the lateral side of the nail bed with the ingrown nail in the great toe showing inflamed medial and lateral gutter and causing redness and tenderness. Huge hypertrophic scar formation secondary to chronic ingrown toe nail mimicking tumor is a rare disease that is not only causing a cosmetic concern, but also hindering normal daily activities physically and socially.

**Intervention and outcomes:**

Excisional biopsy was performed for both great toes. Biopsy confirmed chronic ulcerative inflammation with a hypertrophic scar. The resection site healed and persisted well at 12 months after surgery.

**Conclusion:**

Our unusual case suggests that the natural course of an untreated ingrown toe nail may result in hypertrophic scar extending far to mimic tumorous conditions.

## 1. Introduction

Hypertrophic scar, one of the most common scar types, is caused by disruption of the physiologic wound healing process with repetitive irritation applied to the deep dermis. It is generally has certain predisposing factors such as wound infection. Hypertrophic scar occurs within 4 to 8 weeks following a wound infection or other traumatic skin injuries.^[1,2]^

Hypertrophic scars are contained within the site of injury. They might regress over time. Keloids will spread beyond borders of the initial injury. They do not regress. By definition, a keloid will extend beyond borders of the original scar. Hypertrophic scars have a higher incidence of occurrence than keloids. They remain confined to the original wound border.^[3]^ In contrast to the definition described above, our case presented histologically confirmed hypertrophic scar extending its original scar and presenting in huge size.

A huge hypertrophic scar formation secondary to chronic ingrown toe nail mimicking tumor is a rare disease. It is not only causing concerns cosmetically, but also hindering normal daily activities physically and socially.

In this paper, we present an unusual case of bilateral ingrown nails with different phases. One resulted in a large hypertrophic scar caused by stimulation from secondary to chronic ingrown nail.

### 1.1. Consent

The patient signed informed consent for the publication of this case report and any accompanying images. Ethical approval of this study was waived by the ethics committee of Jeonbuk National University Hospital because it was a case report fewer than 3 patients.

## 2. Case presentation

**Figure 1.**
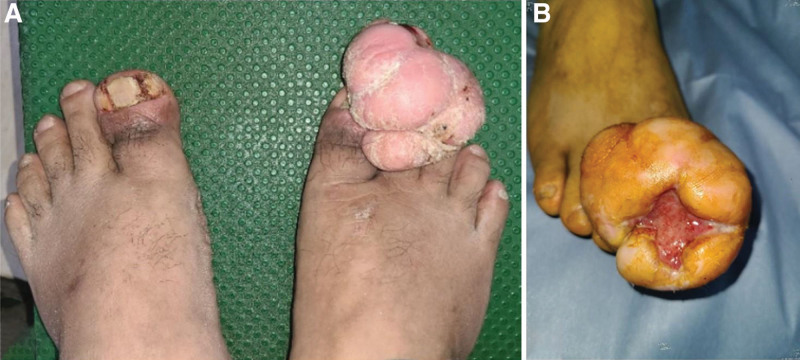
(A) Right great toe lesion with an irregular, bizarre-shaped mass and left great toe lesion with divided nail, inflamed medial and lateral gutter with redness and tenderness; (B) 2 cm × 2 cm sized ulcer at the distal end; (C) Right great toe mass originating from the lateral gutter (7 × 4 × 8.5 cm).

**Figure 2.**
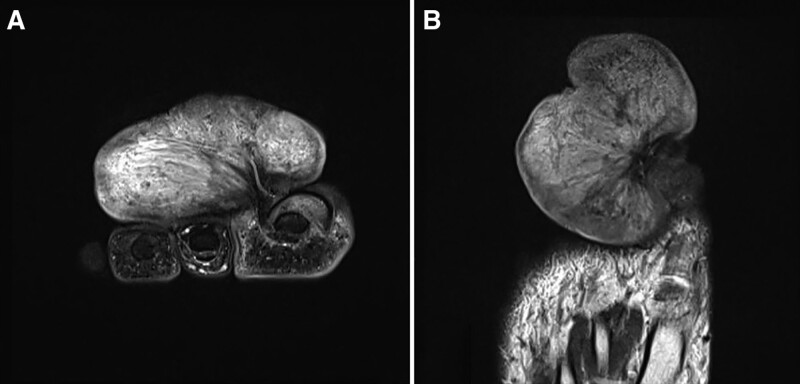
(A) Coronal and (B) axial images of MRI T2WI of the right toe, showing a huge fibrous component soft tissue mass originating from the lateral side of nail bed.

**Figure 3.**
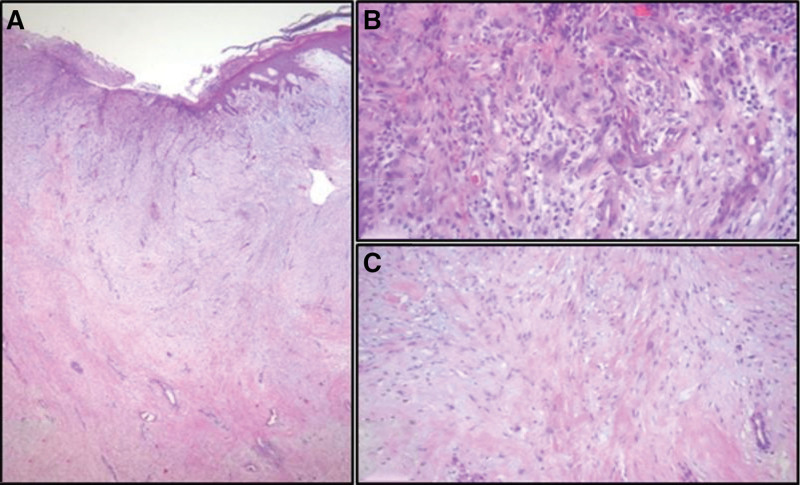
(A) Gross photo of excised mass; and (B) Histologically at low power, the surface of the lesion is ulcerated (arrow) and the adjacent squamous epithelium is hyperplastic. Underneath of ulcer and the hyperplastic squamous epithelium, a of hypertrophic scar composed of the dense collagenous stroma and spindled fibroblasts is found. (C, D) High-power view of the ulcer (B) and hypertrophic scar (C). Original magnification: (A) ×40, (B, C) ×400.

A 44-year-old man with a huge mass (7 × 4 × 8.5 cm) in his right great toe and inflamed ingrown nail in his left great toe visited the clinic. The lesion on the right great toe was an irregular, bizarre-shaped mass with a 2 cm × 2 cm sized ulcer at the distal end (Fig. 1). The inflamed ingrown nail in the left toe showed divided nail with inflamed medial and lateral gutter causing redness and tenderness (Fig. 1). Initial surgery was performed for the right great toe to remove the ingrown nail 3 years ago at another hospital. Since then, he has managed the wound on his own without any treatment from medical professionals. In the process, a tumor-like mass of unknown origin grew gradually and steadily, resulting in intolerable discomfort that caused him to visit the clinic. In gross appearance, a stalk appeared to have grown from the lateral side of the nail bed in the right great toe. No bony invasion was observed on SPECT/CT. MR images showed a huge fibrous component soft tissue mass originating from the lateral side of the nail bed of the right great toe (Fig. 2). Excisional biopsy was performed. Biopsy confirmed chronic ulcerative inflammation active with a hypertrophic scar. Histologically, at the low power field, ulcerated lesions in the superficial layer and hypertrophic adjacent squamous epithelium were observed. Underneath ulcers and proliferative squamous epithelium were characterized by a hypertrophic scar composed of a dense collagenous stroma and spindle-shaped fibroblasts (Fig. 3). After performing surgical excision, adequate wound healing was achieved for both great toes with excellent patient satisfaction. This status was maintained at a 12-month follow-up visit.

## 3. Discussion

There are several types of scar tissues, most notably hypertrophic scar and keloid formation. Histological confirmation is essential because inaccurate diagnosis can lead to inappropriate treatment. In gross appearance, hypertrophic scar may look similar to keloid. It is difficult to differentiate them with naked eyes because a hypertrophic scar can proliferate beyond the expected range. Keloids are genetically predisposed. They can occur anywhere secondary to skin injury. The possibility of a hypertrophic scar exists in cases with overgrown tissues due to continuous irritation or inflammation that develops in an advanced age without the occurrence of similar lesions in other areas.^[4,5]^

In the case report by Lee et al,^[2]^ a case with periungual keloid formation in the left great toe secondary to chronic paronychia and onychocryptosis with a history of previous keloid formation in the left earlobe represented a differential diagnosis to our case. For such a keloid lesion, wedge resection was performed several times. However, it failed. Eventually, the wound improved through tangential excision and multimodal post-operative wound care at 7 years after onset, showing a good prognosis.^[2]^

In addition, hypertrophic scar may present in different morphologies. A previously published report by Samaras et al, a case with a large periungal soft tissue mass associated with onychocryptosis lasting more than 10 years presented similar gross appearance to our case.^[1]^ However, it was different from our case in that our case had ulcer at the tip of the hypertrophic scar. In the abovementioned case, the patient had a large sized mass-like lesion around the ingrown nail without genetic predisposition to keloid formation. After complete excision, nail matrixectomy, and distal tuft resection were performed, satisfactory results were obtained in a long-term follow-up of 5.5 years.^[1]^

Similarly, in the present case, treatment was delayed due to psychological issue of the patient. As a result, a very large mass was generated by repeated inflammation caused by irritation of the ingrowing nail. Ulcerative lesions accompanying the mass continued to grow steadily. Such lesion caused difficulty in wearing shoes or socks. However, treatment was delayed due to personal circumstances because the patient did not feel the need for treatment.

Hypertrophic scar requires differentiation from various periungual forms of tumor-like lesions such as fibrosarcoma^[6]^ and keloid formation. Unlike keloid formation with a strong genetic predisposition, histological confirmation is required for scarring formation that does not have a traumatic event causing keloid formation or occurring at an advanced age or at an uncommon site.^[1]^

Besides keloids, other conditions such as dermatofibroma, dermatofibrosarcoma, desmoid tumor and giant cell tumor must also be considered. Other benign lesions such as epidermal and mucoid cyst may occur. Malignant tumors such as squamous cell carcinoma and malignant melanoma must be excluded as well.^[7–10]^

Although pathologic confirmation is warranted for diagnosis, the mass in our case was huge in gross appearance. However, its slow-growing tendency and homogenous mass without any protuberant nodules or discoloration suggested that it was a benign lesion.

Keloid was excluded from our patient possible diagnosis as he did not have any genetic predisposition nor other previous history of keloids. Other possible benign and malignant lesions mentioned above were excluded from the diagnosis after histologic confirmation.

To the best of our knowledge, previously reported cases showed overgrown hypertrophic scar without ulcerative lesion. Our case shows that ulcerative lesion might form in such a hypertrophic scar which may encourage overgrowth. Our case showed a larger lesion compared to other previously reported cases. The possible cause of such a huge sized mass might be multi-factorial. Ulcerative lesion might have played a key role as repetitive inflammation could continuously stimulate the growth of such lesion in the end. In addition, the natural course of the scarring process is noteworthy. Bilateral ingrown nail in different phase suggests that under the same circumstance, untreated inflamed ingrown nail such as the lesion in the left great toe in our patient might grow continuously to the point mimicking tumorous conditions like the lesion in the right great toe. Accompanying ulcerative lesion might stimulate growth of the mass and the untreated ingrown nail might become hypertrophic scar tissue which may mimic tumor like conditions, suggesting that early appropriate treatment is necessary. Limitations to this study is relatively short follow up period.

## 4. Conclusion

This paper presents the natural course and treatment of hypertrophic scar secondary to continuous irritation caused by a chronic ingrown nail (= onychocryptosis). Scar tissue preventive management was performed after surgical excision. Based on histological diagnosis, it was confirmed that the lesion was benign and differentiated from keloid formation. Satisfactory results were obtained without recurrence during 1 year follow-up. In particular, unlike a previously known characteristic of a hypertrophic scar, not extending beyond its original scar, our case presented a hypertrophic scar with a huge size that outgrew from its original margins. In addition, when comparing the development process of lesions on both sides, preventing lesions might be important as they can continuously and indefinitely enlarge due to various factors such as the environment inducing stimulation or tissue condition (e.g., ulcers) that is prone to inflammation. Therefore, education is essential. If hypertrophic scar has already occurred, appropriate treatment should be given early.

## Author contributions

**Conceptualization:** Kwang-Bok Lee.

**Data curation:** Baruenchan Ju, Young Sang Lee, Dong Hee Kim.

**Funding acquisition:** Kwang-Bok Lee.

**Methodology:** Dong Hee Kim.

**Writing – original draft:** Baruenchan Ju, Young Sang Lee.

**Writing – review & editing:** Kwang-Bok Lee.
